# A compact setup for behavioral studies measuring limb acceleration

**DOI:** 10.1016/j.ohx.2024.e00522

**Published:** 2024-04-06

**Authors:** J. Rapp, B. Sandurkov, P. Müller, N.H. Jung, B. Gleich

**Affiliations:** a Munich Institute of Biomedical Engineering (MIBE), Technische Universität München, Garching 85748, Germany; b Department of Pediatrics, Technical University Munich, Kinderzentrum München gemeinnützige GmbH, Heiglhofstrasse 65, Munich 81377, Germany

**Keywords:** Acceleration, Thumb movement, Neural plasticity

## Abstract

Behavioral studies contribute largely to a broader understanding of human brain mechanisms and the process of learning and memory. An established method to quantify motor learning is the analysis of thumb activity. In combination with brain stimulation, the effect of various treatments on neural plasticity and motor learning can be assessed. So far, the setups for thumb abduction measurements employed consist of bulky amplifiers and digital-to-analog devices to record the data. We developed a compact hardware setup to measure acceleration data which can be integrated into a wearable, including a sensor board and a microcontroller board which can be connected to a PC via USB. Additionally, we provide two software packages including graphical user interfaces, one to communicate with the hardware and one to evaluate and process the data. This work demonstrates the construction and application of our setup at the example of thumb acceleration measurement with a custom made glove and its use for research. Using integrated circuits, the size of the measurement devices is reduced to this wearable. It is simple to construct and can be operated easily by non-technical staff.

## Graphical abstract

**Figure:**
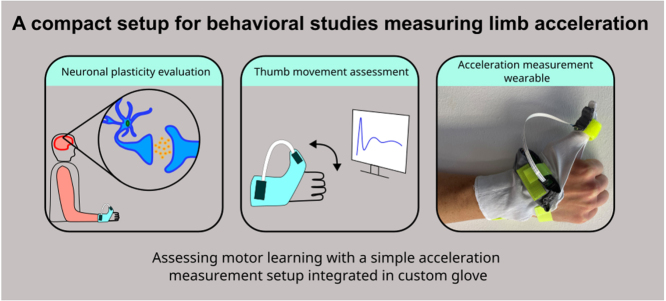

## Specifications table

**Table:** 

Hardware name	*Glove integrated thumb acceleration measurement*
Subject area	•*Engineering and material science*
Hardware type	•*Measuring physical properties and in-lab sensors*
Closest commercial analog	*No commercial analog is available.*
Open source license	*CERN-OHL-W-2.0*
Cost of hardware	*150 €*
Source file repository	https://doi.org/10.5281/zenodo.10014129
OSHWA certification UID	*DE000141*

## 1 Hardware in context

A solid understanding of learning and memory processes in the human brain is a desirable goal for many researchers. Motor learning is subject of considerable interest, since it is crucial to acquire new motor skills or regain movement ability after loss of function due to stroke or other neurological diseases. Thus, neural plasticity, acting as basis for memory and learning, has been in the focus of several studies [1], [2], [3]. Especially, the impact of long-term potentiation (LTP) is considered to play a crucial role in motor learning [4]. However, several mechanisms of neural plasticity in motor cortical brain structures are not fully understood so far [2]. A popular method for gaining a deeper understanding of such brain structures is the stimulation of according areas, for example by using transcranial magnetic stimulation, and observing changes in behavior and neural plasticity. Several experimental protocols have been tested to evaluate the effect of stimulation patterns. Further research has been conducted on the impact of exercise or nicotine on neural plasticity [5]. Opie et al. could show that exercise results in improved ballistic performance [6]. To quantify motor learning, the assessment of motor practice has been established as a useful method. The before mentioned studies conducted thumb abduction tasks to measure the subject’s performance. Using accelerometers, the change of peak acceleration after stimulation has been used as an indicator for motor practice.

Usually, such a setup consists of an accelerometer, an amplifier system and digital-to-analog conversion [2], [3], [5]. So far, each of those components has been selected individually and the setup was assembled to a bulky combination of line power dependent devices. No specification was given for the amplifiers either. Those inappropriate and complicated setups lead to inflexible experiments and usually require not only medical staff, but also engineers to operate.

Several studies have already investigated limb movement using body-attached accelerometer data [7], [8], [9]. However, those previous studies focus rather on quantitative measurements than on the precise evaluation of acceleration. Additionally, for the experimental purposes described above, we were able to further reduce the complexity compared to the approaches of [7], [8], [9], where motion capture or other quantities are used.

In our approach, we combined an acceleration sensor with a compact microcontroller board which can be connected to a laptop or PC. A buzzer is integrated to output an audio signal which triggers the subject to initiate the movement. With simple schematics and commonly available components, our open source setup is a cheap and lightweight alternative. Additionally, we provide a fast and simple open source software called *Accelera*, implemented in C# including a graphical user interface (GUI) to operate the device and analyze the data in real time. The GUI contains an adaptable measurement protocol to reduce the organization workload during the experiment. To further analyze and extract the relevant data from the recorded movement, we present the open source software *Viewcelera*.

Our setup can be used arbitrarily at any body part. As a demonstration, we adapted it for the thumb abduction task and integrated the hardware in to a custom designed glove. Slight modifications of the wearable make it transferable to similar applications at other limbs. With our adapted and optimized all-in-one device we hope to simplify experiments recording limb acceleration, for example in combination with magnetic stimulation. We expect to enable a broader range of experimental applications, due to less complex setups. Simple staff can operate the compact device without the help of engineers or external expertise.

## 2 Hardware description

Our device is implemented as a wearable to optimize patient comfort and experimental procedures. An acceleration sensor and the corresponding hardware are integrated into a glove which can be connected to a PC via USB cable. The setup can operate at sampling rates between 4 Hz and 4 kHz. To operate the sensor and conduct the experiments, we developed the open source software *Accelera*. For data processing and analysis, we provide *Viewcelera*. Both are easy to use and include a GUI.

### 2.1 Sensor

Nowadays, with the use of micro-electro-mechanical-system (MEMS) sensors, 3-axis accelerometers are available for a broad range of applications. Crucial parameters are the range (usually given as a multiple of the gravitational constant), bandwidth, data rate and the spatial direction of measurement. For thumb abduction tasks, we chose the ADXL355 (Analog Devices, Massachusetts, USA), a sensor with a range of a maximum of 8 G, 3 axes and low noise. For the sensor we designed a small printed circuit board (PCB) of two layers and a size of 15 mm × 19 mm which can be connected to the microcontroller board.

### 2.2 PCB and microcontroller

For minimum size and efficient data transfer we designed a custom PCB including an ST Cortex F4 microcontroller (STM32F401RET6, STMicroelectronics, Switzerland). The controller board collects and computes the sensor data and transfers it via USB to a PC. Further, a 4 kHz piezo buzzer (PKLCS1212E4001, Murata, Japan) is used to output the audio signal for the subject to indicate the thumb movement. At this time point, the data recording is started. Alternatively, an external trigger can be used which can be relevant for experiments were the subject is stimulated with magnetic stimulation. In such a case, the recording of the sensor can be started in relation to the magnetic stimulation pulse. The circuit has an edge-controlled trigger input (connector X3 in the schematics). It can be configured in *Accelera*, such that a measurement can be started on either rising or falling edge. The input is 5 V tolerant. A logical high is guaranteed to be detected from a level greater than 1.785 V. A logic low is guaranteed from a level of less than 1.115 V. With a size of 35 mm × 55 mm, the board completes the compact design of the setup. The controller PCB consists of 4 layers, two signal layers on top and bottom, a ground plane and one plane for 3.3 V supply. It contains the microcontroller, a voltage converter for the component’s power supply, the buzzer, an USB connector and an FFC connector for the sensor board. The microcontroller can be configured using *Accelera*. A firmware for the microcontroller is provided as well.

Future improvements of this setup could include wireless data transmission between controller board and PC.

### 2.3 *Accelera*

Instead of formerly required licenced recording and signal processing software like SIGNAL (Cambridge Electronic Design Ltd, Cambridge, UK) [1], [2], we provide an open source user interface called *Accelera* to collect and display the acceleration data. *Accelera* allows the user to setup complete measurement protocols including adaptable intervals for stimulation and recording [1], [3]. For clarity, we introduce the terms “sample” for the acceleration at each time point, “event” for one second of recording after the trigger and “block” for one session of events separated by the breaks. For further analysis, the data is stored in three text files. The first file contains the acceleration data in all directions as well as the ID for each sample, event and block. Raw data and sensor temperature are stored there as well. The second file contains real time stamps for each sample monitor breaks and delays. The third file provides meta information, such as subject information, a summary of the event and block numbers, and the hardware configuration. *Accelera* can be used to update the microcontroller with the desired protocol to adapt recording times, intervals between blocks and events and configuration of the trigger mode. These features allow an operation of the setup by technically inexperienced staff. *Accelera* is implemented in C#.

### 2.4 *Viewcelera*

Evaluating the recorded data can be done using MATLAB (The MathWorks, Inc., USA), Python or similar tools. Due to the sophisticated protocols, the proper interpretation of the data is complex. Further, the movement of the subject within the recorded timespan varies between the samples. Hence, we provide *Viewcelera* which allows to examine the data sets and manually select the relevant movement samples from the recorded data within an event and perform statistical analysis. The statistically relevant values can be exported as a text file. *Viewcelera* is implemented in C++ and Qt (The Qt Company, Espoo, Finland) for fast and efficient GUI performance.

### 2.5 Glove

**Fig. 1:**
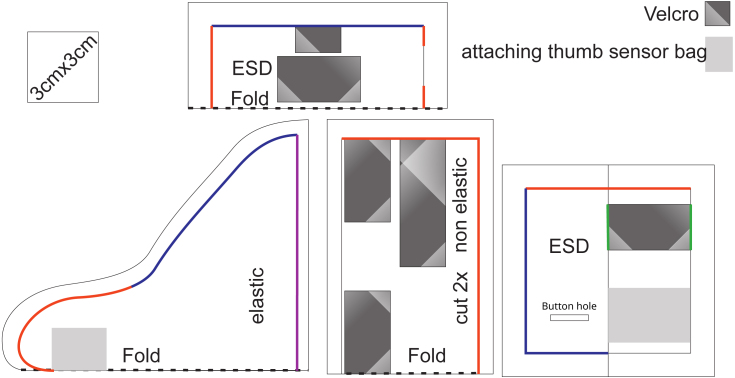
Cutting pattern of the glove; The materials are listed in the bill of material table. (For interpretation of the references to color in this figure legend, the reader is referred to the web version of this article.)

Wearables are a convenient method to fix electrical components to human bodies. Alternatives like taping are not only less comfortable but can also damage the hardware. To save space and reduce the costs of production, we decided against a housing for the electronics and chose electrostatic discharge (ESD) proof fabric instead. The glove comes with a Velcro fastener for adaption to different sizes. A cutting pattern is provided in Fig. 1. The glove can easily be adapted for the application on other fingers.

### 2.6 Summary

• *Compact wearable to measure sensible accelerometer data of fingers*
• *No extra hardware, can be connected to PC*
• *Easily adaptable for other body parts or objects*
• *Custom made software *Accelera* to operate sensor and conduct experiments*
• *Graphical interface *Viewcelera* to analyze data*

## 3 Design files summary

All design files, including the code for firmware and other software, can be found in the source file repository ( https://doi.org/10.5281/zenodo.10014129). The circuits and PCB layout for the hardware are provided additionally in the supplementary material.

**Table:** 

Design file name	File type	Open source license	Location of the file
Hardware-1.zip	PDF and Altium Project	CERN-OHL-W-2.0	https://doi.org/10.5281/zenodo.10014129
Firmware-1.zip	Firmware for Controller board	CERN-OHL-W-2.0	https://doi.org/10.5281/zenodo.10014129
Accelera-1.zip	Visual Studio Project	CERN-OHL-W-2.0	https://doi.org/10.5281/zenodo.10014129
Viewccelera-1.zip	Qt Project	CERN-OHL-W-2.0	https://doi.org/10.5281/zenodo.10014129

## 4 Bill of materials summary

A complete list of the components and materials used can be found in the supplementary material.

## 5 Build instructions

This chapter describes the cutting instructions for the textiles of the glove, the manufacturing of the PCB layout, the proper connection of all components and the operation of *Accelera* and *Viewcelera*.

### 5.1 Required tools

To fabricate the glove, a cutting knife and sewing equipment is needed. If modifications of the PCB are desired, Altium Designer (Altium Ltd., USA) is required. For adaptions or compiling of *Viewcelera*, a Qt compiler is needed. A For the fabrication of the PCB, we recommend to send the files to a qualified manufacturer. The electrical components can be placed onto the PCB using soldering equipment or ideally, a pick and place machine. To operate *Accelera* and *Viewcelera*, a Windows PC is required. In order to program the microcontroller, a programming adapter with associated software is required. Suitable programming adapters would be STLink V2 (ST Microelectronics, Plan-les-Ouates, Switzerland), ULINK2 (Keil, Munich, Germany) or the J-Link (Segger, Mohnheim am Rhein, Germany).

### 5.2 Sewing of the glove

The textiles in the following fabrication manual are marked with numbers corresponding to the designator in the bill of material. For the thumb sensor bag, use the antistatic fiber (1). First, sew the buttonhole in the thumb pocket, according to Fig. 1, then overcast along the blue marking and close along the red marking. Take 10 cm of both types of Velcro and sew them together back to back, then sew this stripe to the marking on the bag along the green markings. The elastic thumb section is made with the elastic fiber (2). The thumb part is closed along the red mask, hemmed along the blue mark. Sew the thumb sensor bag to the thumb section. A non-elastic fabric (3) is required for the wrist section. Place the two parts with each right side close along the red marking. Turn the right side out. Add a peace of Velcro loop on the upper marking and another Velcro loop on one side of the lower marking. Add a long piece of Velcro hook tape on the other side. Fold the seam allowance inside and place the purple marking in between the layers and sew it shut.

### 5.3 Printed circuit board (PCB)

**Fig. 2:**
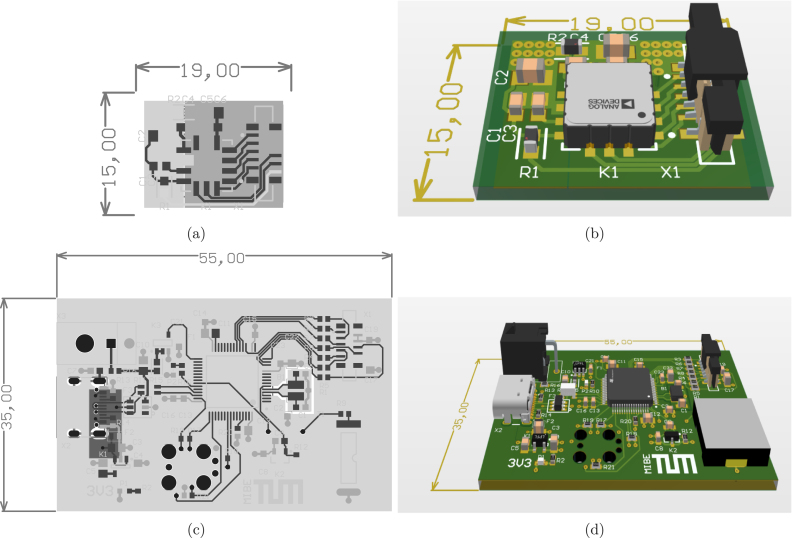
(a) PCB layout of the sensor board; (b) 3D model of the sensor board; (c) PCB layout of the controller board; (d) 3D model of the controller board.

The electrical components are placed on two PCBs, the sensor board and the controller board. Circuit schematics and layout can be found in the supplementary design files. The layouts can be used to manufacture the circuit board, which is usually done by common circuit board services, like PCB Pool (Aarbergen, Germany). Modifications of the circuit board can be done by loading schematics and layout into Altium Designer. Alternatively, the provided circuit must be inserted in a equivalent electronic design automation software like KiCad (GNU General Public License). The PCB layouts for the sensor boards and the controller board are shown in Fig. 2.

### 5.4 PCB assembling

**Fig. 3:**
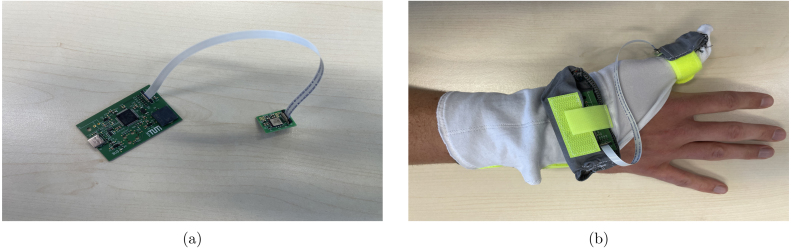
(a) The sensor board connected to the microcontroller board; (b) The glove applied to a wrist with the two PCBs in the accordingly attached bags.

Once the PCB is manufactured, the electrical components must be soldered to their assigned positions. A list of all required components is in the bills of material provided in the supplementary files. The designators of the components are printed on the surface of the PCB to indicate where each component has to be placed. To save space, we used surface mounted components only. Ideally, a pick and place machine and a reflow oven or similar assistance is used to solder the components. For experienced people, manual soldering is possible, alternatively, many of the above mentioned circuit board services offer assembling as well. The connector for the trigger input is optional and was not placed on the board shown in Fig. 3. Fig. 2 shows the board including the connector for the trigger.

### 5.5 Mounting of the setup

To connect sensor and controller board a flat ribbon cable is used (see Fig. 3). For the circuit boards, there are two bags in the glove. The controller board is inserted into the larger bag at the ball of the hand. The sensor board is inserted into the smaller bag at the tip of the thumb such that the bottom of the board lies on the thumb and the cable points outwards. Thereby, the board is positioned in a way that the flat ribbon connector is at the opposite of the thumb tip, as shown in Fig. 3. Generally, the position can be modified but should be kept constant during the experiments.

### 5.6 Software installation

Finally, to take the device into operation, the software must be installed. For the setup of *Accelera* and *Viewcelera* in Windows, an exe-file is provided. To operate the hardware, we provide a firmware for the microcontroller, which must be flashed to the microcontroller with an arbitrary programmer via the Plug-of-nails connector.

## 6 Operation instructions

To collect data from the sensor, or execute experimental protocols, *Accelera* must started on a PC. The glove is applied to the subject’s hand. Sensor and the controller board are inserted into the corresponding bag of the glove and connected with the flat ribbon cable. A picture of the two connected PCBs and the whole setup including the glove is shown in Fig. 3. The controller board must be connected to the PC with an USB-C cable.

**Fig. 4:**
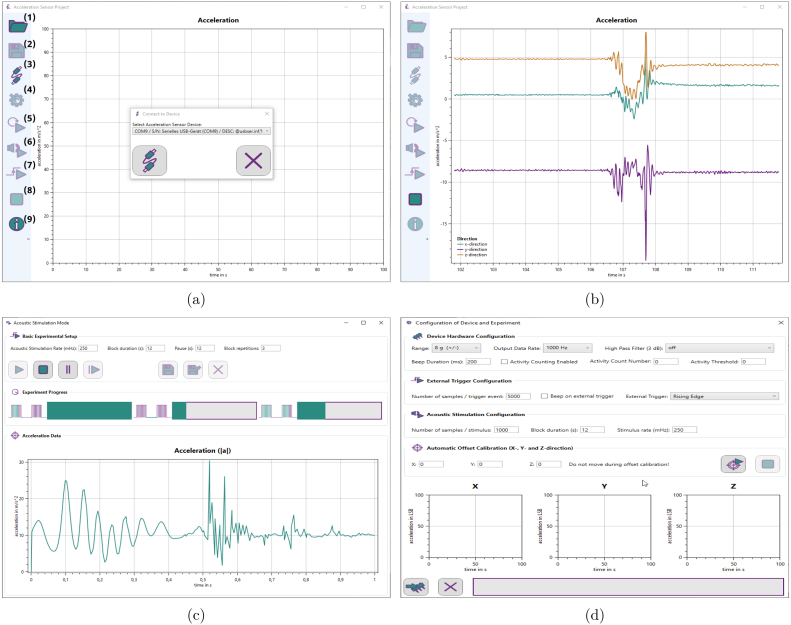
*Accelera* screenshots: (a) Connecting *Accelera* to the microcontroller board; The numbers are labeling the available buttons: (1) open a previously stored file; (2) Save recorded data to file; (3) connect a hardware device; (4) options, the corresponding widget is shown in (d); (5) record data in continuous mode; (6) start protocol mode; (7) start external trigger mode; (8) stop continuous mode; (9) software information; (b) Data visualization of *Accelera* in continuous mode; (c) Protocol mode with three sections; Basic Experimental Setup: allows the user to set the timings for the protocol; Experiment Progress: Shows the progress of the current experiment block, the progress of the break and the progress of the whole experiment; Acceleration Data: shows the currently recorded curve; (d) The settings window can be used to configure the hardware and calibrate the sensor.

### 6.1 Manual for *Accelera*

Screenshots of *Accelera* and enumerated buttons are shown in Fig. 4. The first button “Open” (1) allows the user to open a formerly stored data set for reviewing. To establish a connection to the hardware, the “Connect device” button (3) must be pressed and the device selected from a list in the connection window. With the device being ready, three modes can be selected in the next step: Free running mode (5), acoustic stimulation mode (6), external trigger mode (7).

#### 6.1.1 Free running mode

If this mode is selected, the device starts measuring and the acceleration in x, y and z direction as well as the absolute value is displayed live in the *Accelera* window. The measurement can be terminated by pressing the “Stop” button (8). Subsequently, the recorded data can be stored into a file by pressing the “Save” button (2).

#### 6.1.2 Acoustic stimulation mode

Entering the acoustic stimulation mode, which is specifically designed for sophisticated experimental protocols, will open another window. A screenshot is provided in Fig. 4. The user can select the acoustic stimulation rate (beeping sound) in mHz, the block duration in s, the pause between the blocks in s, and the block repetitions. There are buttons to start the experiment, pause it and stop it. After pressing the stop button or finishing the experiment, the data can be stored to a file. There are two saving modes: one that simply saves the data and one that allows the user to add subject information. Additionally, the acoustic stimulation window shows progress bars for the blocks, the pauses and the whole experiment and an acceleration plot displaying the current event (in x, y, z direction and absolute value). The implemented protocols are as described in [1], [3]. Statistical comparisons of the acceleration curves over a sufficiently long period allows the evaluation of quantities such as plasticity or the influence of certain factors on learning.

#### 6.1.3 External trigger mode

In the external trigger mode, the data recording is triggered from an external device. After the trigger, the specified number of samples is recorded. After termination of the experiment, the data can be stored into a file.

#### 6.1.4 Settings

The settings window can be opened by pressing the “Setup and settings” button (4). A screenshot is provided in Fig. 4. It provides the options to configure the settings of the hardware, like beeping sound and sample rate. In the first section, “Device Hardware Configuration”, the user can specify the acceleration range in g, the sampling rate in Hz and optionally a high pass filter for the data. Further, the beeping sound duration and an activity count can be specified. In section “External Trigger Configuration”, the number of samples after the trigger event can be set and whether there should be a beep. The trigger signal, falling or rising edge, can be adapted to the requirements of the external device. The “Acoustic Stimulation Configuration” section allows to set the number of recorded samples after the stimulus, the duration of the blocks and the rate of the stimulus in mHz. Section “Automatic Offset Calibration” provides the option to detect offsets in all directions and eliminate them by calibration. Finally, the specified settings can be flashed to the hardware.

### 6.2 Manual for *Viewcelera*

Manual for Viewcelera is detailed here.

### 6.3 Overview

**Fig. 5:**
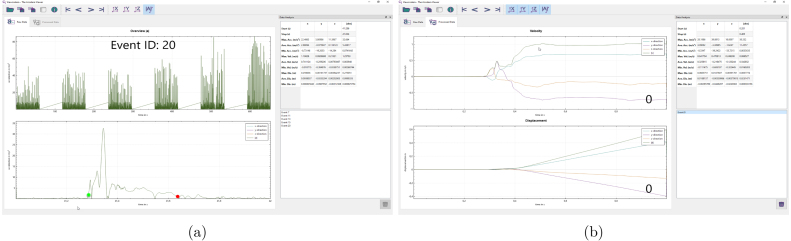
(a) Screenshots of Viewcelera are shown. The upper picture displays data from the whole experiments and the lower plot shows a single event (e.g. #20). Using the mouse, the user can select the relevant data, marked as a green and a red dot. On the upper right, minima, maxima and mean are given for acceleration, velocity and displacement. All data is calculated for x, y and z direction and the absolute value. At the lower right corner, the events where a selection was made are listed and ready for further analysis. (b) Once the relevant data was selected in an event, it can be selected to display velocity and displacement in a new tab. (For interpretation of the references to color in this figure legend, the reader is referred to the web version of this article.)

To extract the relevant information from the recorded protocol data, *Viewcelera* can be used (a screenshot is shown in Fig. 5). It provides an overview over the whole experiment and allows the user to switch between the individual events (recorded data after the trigger). Files previously generated with *Accelera* can be loaded by clicking the “Open” button. After the file has been loaded, the whole experiment is displayed in the upper plot and a single event in the lower plot (see Fig. 5). Using the arrow buttons at the top of the window, the user can switch between the events which are shown in the lower plot. Next to the arrow buttons, there are buttons to select which component of the data is shown, x, y, z direction and absolute value.

### 6.4 Select relevant data

For each event, the relevant acceleration data can be selected by clicking into the plot. The start and end point are indicated with a green and red dot (Fig. 5). Once the relevant data is selected for an event, the event data is put onto a stack of analyzed events, which is shown in the list view at the lower right corner. For the analyzed events, the minima, maxima and means are determined for acceleration, velocity and displacement. Those informations are shown in the widget at the upper right corner. *Viewcelera* shows velocity and displacement in another tab, once an event is selected. The selection of each event can be deleted and redone. This data can be exported for further statistical analysis.

## 7 Validation and characterization

We demonstrate the use of our setup in two experiments. First, we show that the data recorded by the sensor is reasonable. Secondly, we show a typical application with four subjects. The sensor was operated at a sampling frequency of 1 kHz.

### 7.1 Sensor performance

**Fig. 6:**
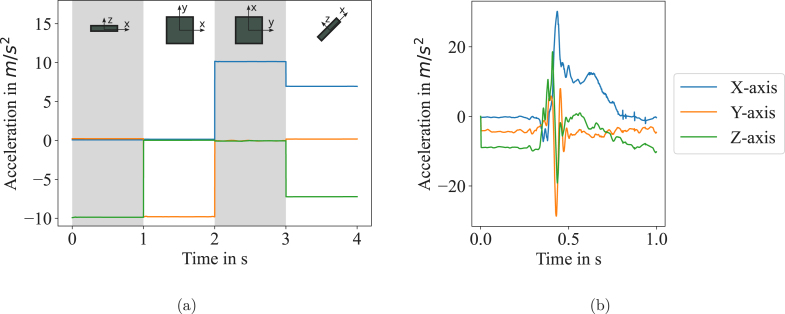
(a) The plots show the acceleration curves after the sensor has been turned in each direction. The directional acceleration component is at 1 g (=9.81 m s^−2^) when the corresponding sensor axis is placed vertically. At 45°, two components are involved. (b) The data shows a single thumb bending experiment. The thumb was in idle position and after a beeping sound the thumb was bent. The legend on the right side is valid for both plots.

To proof the functionality of the setup, we performed some basic experiment types. First, we turned the sensor in all directions such that each axis is placed vertically and recorded the data with *Accelera*. The sensor positions and a plot of the data are shown in Fig. 6. It demonstrates how the gravitation force is shifted between the three axes while the sensor is turned. When one of the sensor axes (x, y, z) is placed vertically, the according acceleration curve of that direction is at 1 g, which corresponds to 9.81 m s^−2^. At a 45° placement, the two involved directional acceleration components are at 6.94 m s^−2^, which again gives 1 g using the Pythagorean theorem. Secondly, we performed a single thumb bending experiment, as it would be carried out in clinical studies. The glove was applied to the subject’s hand, and the whole setup was connected as described above. After the first beeping sound, the subject bent the thumb from idle position into a bent position. The resulting curve is shown in Fig. 6 and demonstrates how the three directional acceleration components contribute to the movement.

### 7.2 Experimental example

#### 7.2.1 Arrangement

To prove suitability of the setup for clinical studies, we conducted experiments on four subjects similar to the protocol design in Teo et al. [5]. The subjects were seated comfortably and put on the glove including the setup as described above. To maintain consistency, the subject’s arm was put on the chair armrest and the thumb pointed upwards. The subjects were told to bend their thumb when they hear the beeping sound. Two subjects were motivated by the examiner during the session whereas there was no motivation in the other two experiments. The protocol consists of six blocks with each 30 recording events of 1000 samples (1 s). Between the blocks was a break of 60 s.

#### 7.2.2 Results

**Fig. 7:**
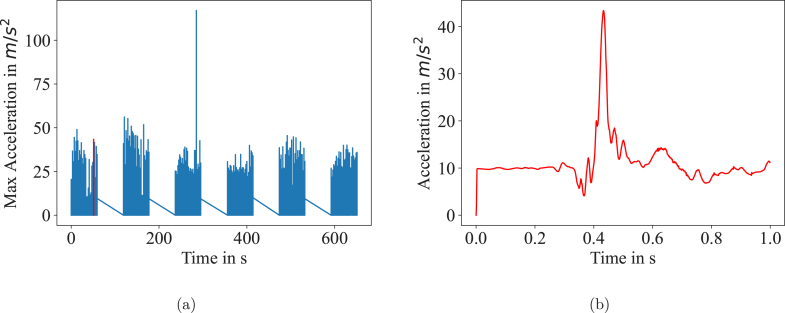
(a) Results of an experimental protocol over six blocks is shown. Each block contains 30 events of 1000 samples. One exemplary event is highlighted in red and plotted again in (b). (For interpretation of the references to color in this figure legend, the reader is referred to the web version of this article.)

The data from one experiment is shown exemplary in Fig. 7. The distinct blocks separated by breaks are clearly visible. One event is highlighted and plotted as well on a distinct time scale.

**Fig. 8:**
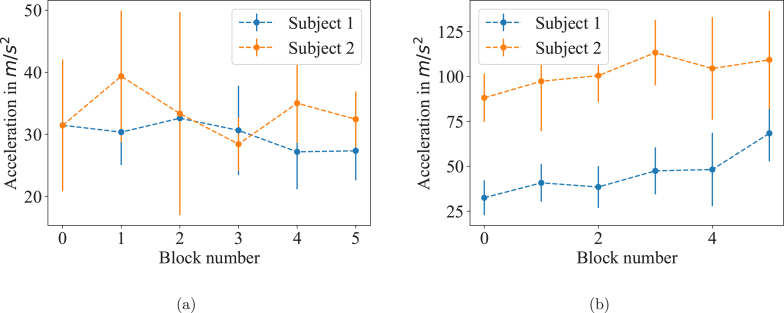
We tested our setup with a protocol of six blocks with each 30 recording events of 1000 samples. Between each block was a break of 60 s. The peak acceleration was determined for each event and the graphs show the mean of the peak acceleration per block. The error bars show the standard deviation. (a) In two experiments, the subjects did not receive any motivation. (b) When the subjects were motivated by the examiner, the peak acceleration increased for each block.

Fig. 8 show the data for these experiments. For each event, the peak acceleration was determined and the average peak acceleration of each block is shown together with the standard deviation in the plots. In comparison to the first block, the last block has a 52.5 % and 18.7 % higher peak acceleration for the two experiments where the subjects were motivated during the session. No increase (-12.6 % and 2.5 %) could be seen in experiment without any motivation by the examiner. These results confirm former investigations [1] and show that motivation plays a crucial role in motor practice. With our experiments we do not intend any conclusions about motor learning but rather show that our setup is suitable to detect relevant acceleration parameters for behavioral research studies. Since the experiments are simply exemplary, no significance tests have been performed on the data, the error bars in Fig. 8 show the standard deviation.

#### 7.2.3 Conclusion

We demonstrated the setup works correctly and is able detect thumb acceleration with high precision. The whole protocol was successfully conducted for each participant and the data showed reasonable results. Further, our software packages have been evaluated. *Accelera* is suitable to record and save the data which is then further processed with *Viewcelera* for statistical analysis, as shown in Fig. 8. From each event, *Viewcelera* was used to select the relevant acceleration data using the start and end point function. Mean and standard deviation are calculated by *Viewcelera*, as well as velocity and displacement.

**Ethics statements**

***This study was approved by the local Ethics Committee (vote 5423/12). After full disclosure of the purpose and risks of the study procedure, all subjects gave their written informed consent***

## CRediT authorship contribution statement

**J. Rapp:** Writing – review & editing, Writing – original draft, Visualization, Formal analysis, Data curation. **B. Sandurkov:** Validation, Methodology, Investigation. **P. Müller:** Validation, Methodology, Data curation. **N.H. Jung:** Validation, Supervision, Methodology, Investigation, Conceptualization. **B. Gleich:** Supervision, Software, Project administration, Methodology, Investigation, Funding acquisition, Conceptualization.

## Declaration of competing interest

The authors declare the following financial interests/personal relationships which may be considered as potential competing interests: Jonathan Rapp reports financial support was provided by German Research Foundation. If there are other authors, they declare that they have no known competing financial interests or personal relationships that could have appeared to influence the work reported in this paper.

## Appendix A Supplementary data

MMC S1 The supplementary contains electrical schematics and layout for the sensor and controller board as well as the bills of material.
