# Supplementary material for: A compact setup for behavioral studies measuring limb acceleration
Source: HardwareX. 2024 Apr 6;18:e00522. doi: 10.1016/j.ohx.2024.e00522 (PMC11022083; doi:10.1016/j.ohx.2024.e00522)
Supplement: MMC S1 — The supplementary contains electrical schematics and layout for the sensor and controller board as well as the bills of material. [file mmc1.zip › OHX_522 - Supplementary files.zip!/OHX_522 - Supplementary files/CTRL_Board_circuit_and_layout.pdf]

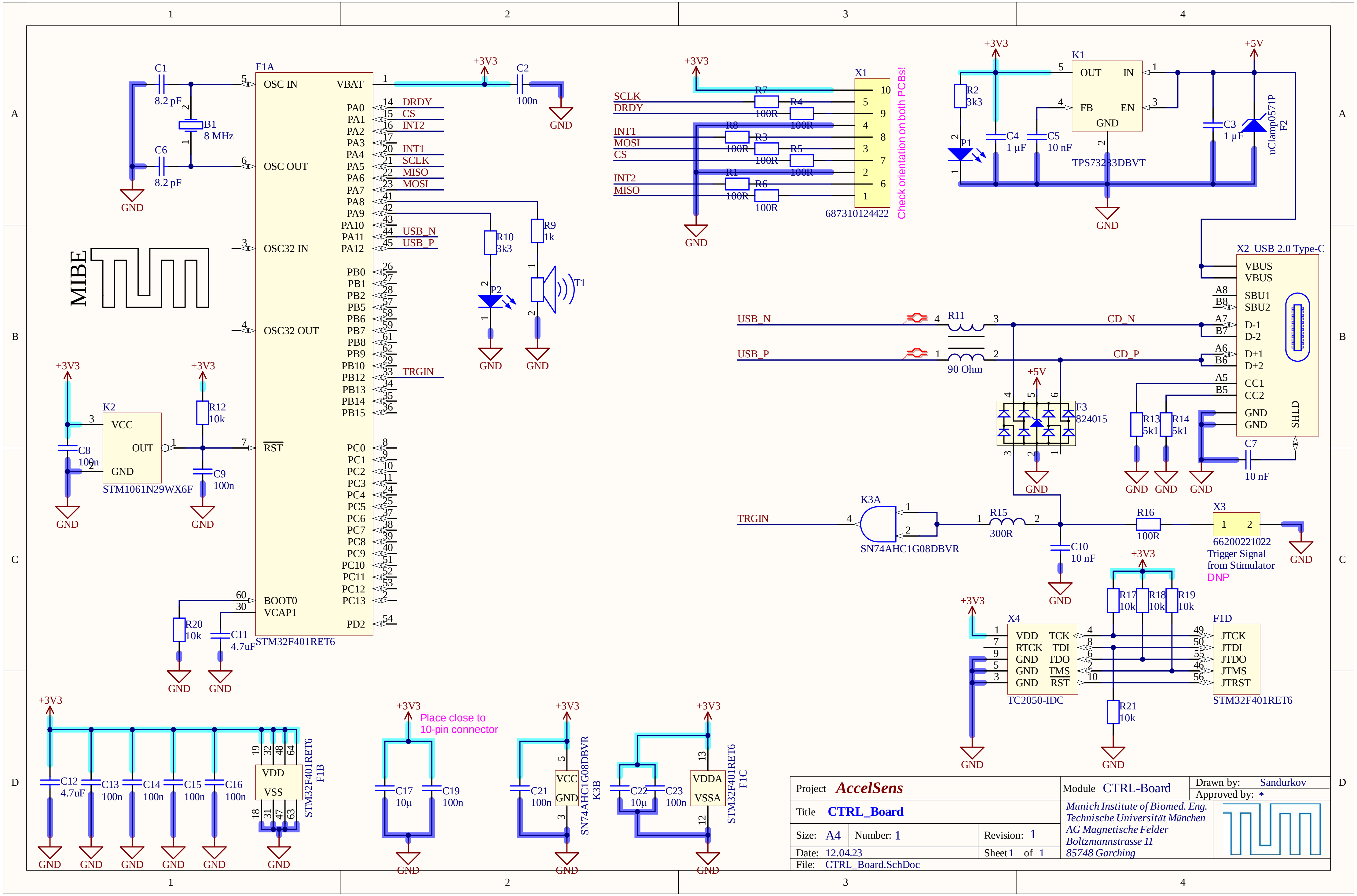
MIBE
F1A
STM32F401RET6
OSC IN
OSC OUT
OSC32 IN
OSC32 OUT
RST
BOOT0
VCAP1
VBAT
DRDY
CS
INT1
INT2
SCLK
MISO
MOSI
USB_N
USB_P
TRGIN
+3V3
+5V
GND
C1 8.2 pF
C6 8.2 pF
B1 8 MHz
C2 100n
R10 3k3
R9 1k
P2
T1
K2 STM1061N29WX6F
VCC
OUT
R12 10k
C8 100n
C9 100n
R20 10k
C11 4.7uF
X1 687310124422
Check orientation on both PCBs!
R1 R3 R4 R5 R6 R7 R8 100R
K1 TPS73233DBVT
OUT IN FB EN
R2 3k3
P1
C4 1 µF
C5 10 nF
C3 1 µF
uClamp0571P F2
X2 USB 2.0 Type-C
VBUS
SBU1
SBU2
D-1
D-2
D+1
D+2
CC1
CC2
SHLD
CD_N
CD_P
R11 90 Ohm
F3 824015
R13 5k1
R14 5k1
C7 10 nF
K3A SN74AHC1G08DBVR
R15 300R
R16 100R
C10 10 nF
X3 66200221022
Trigger Signal from Stimulator
DNP
R17 10k
R18 10k
R19 10k
R21 10k
X4 TC2050-IDC
VDD RTCK GND TCK TDI TDO TMS RST
F1D STM32F401RET6
JTCK JTDI JTDO JTMS JTRST
C12 4.7uF
C13 100n
C14 100n
C15 100n
C16 100n
F1B STM32F401RET6
VDD VSS
Place close to 10-pin connector
C17 10µ
C19 100n
C21 100n
K3B SN74AHC1G08DBVR
C22 10µ
C23 100n
F1C STM32F401RET6
VDDA VSSA
Project AccelSens
Module CTRL-Board
Drawn by: Sandurkov
Approved by: *
Title CTRL_Board
Size: A4
Number: 1
Revision: 1
Date: 12.04.23
Sheet 1 of 1
File: CTRL_Board.SchDoc
Munich Institute of Biomed. Eng.
Technische Universität München
AG Magnetische Felder
Boltzmannstrasse 11
85748 Garching

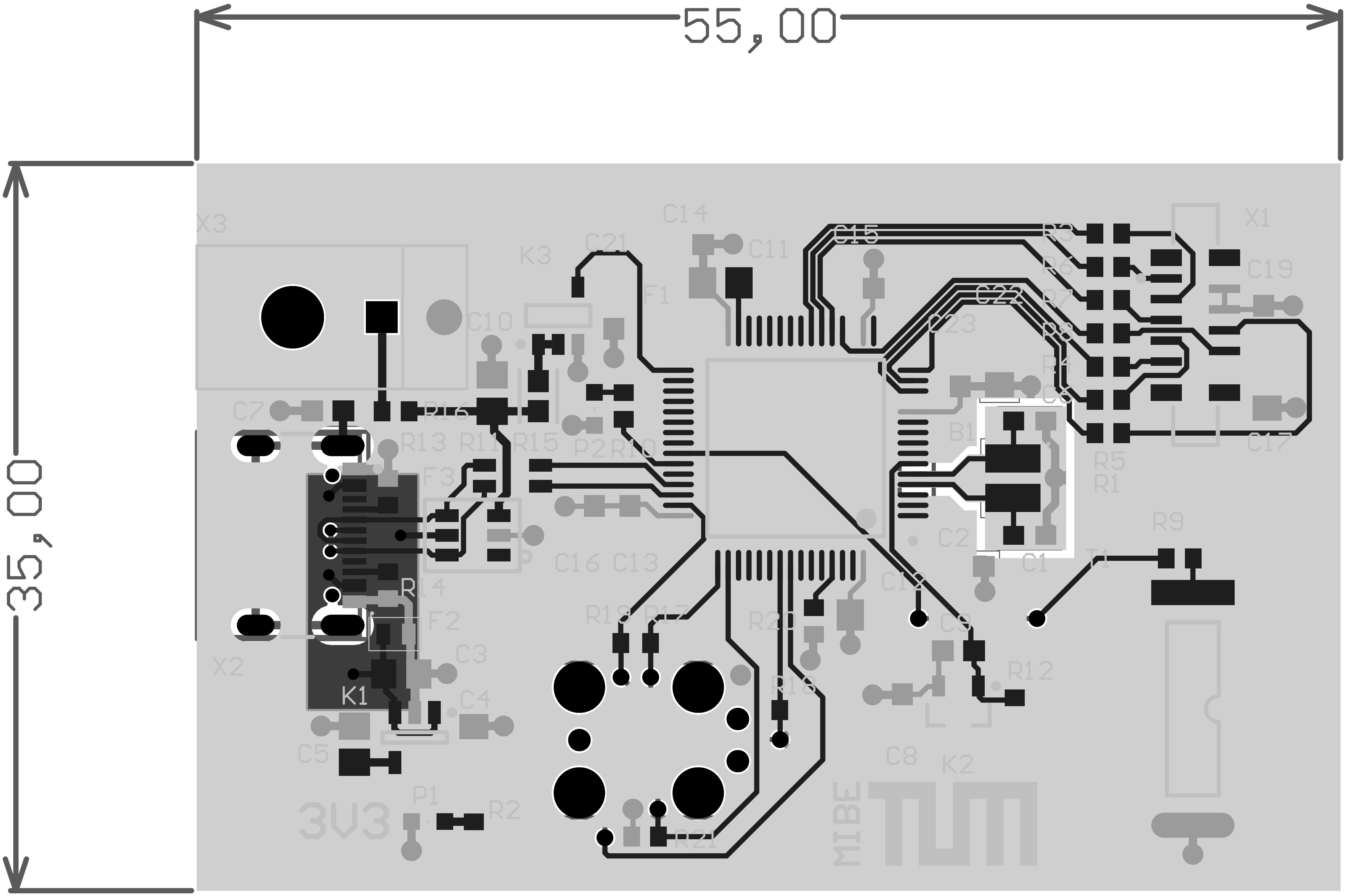
55,00
35,00
X3
K3
C14
C21
C11
C15
X1
R3
R6
R7
R8
R4
R5
R1
C19
C22
C23
C10
F1
C7
R16
R13
R11
R15
P2
R10
C6
B1
C17
F3
C16
C13
R9
C2
C1
T1
R14
C12
F2
R19
R17
R20
C9
X2
C3
R12
K1
C4
R18
C5
C8
K2
P1
R2
3V3
R21
MIBE
