# Supplementary material for: A compact setup for behavioral studies measuring limb acceleration
Source: HardwareX. 2024 Apr 6;18:e00522. doi: 10.1016/j.ohx.2024.e00522 (PMC11022083; doi:10.1016/j.ohx.2024.e00522)
Supplement: MMC S1 — The supplementary contains electrical schematics and layout for the sensor and controller board as well as the bills of material. [file mmc1.zip › OHX_522 - Supplementary files.zip!/OHX_522 - Supplementary files/SENSOR_Board_circuit_and_layout.pdf]

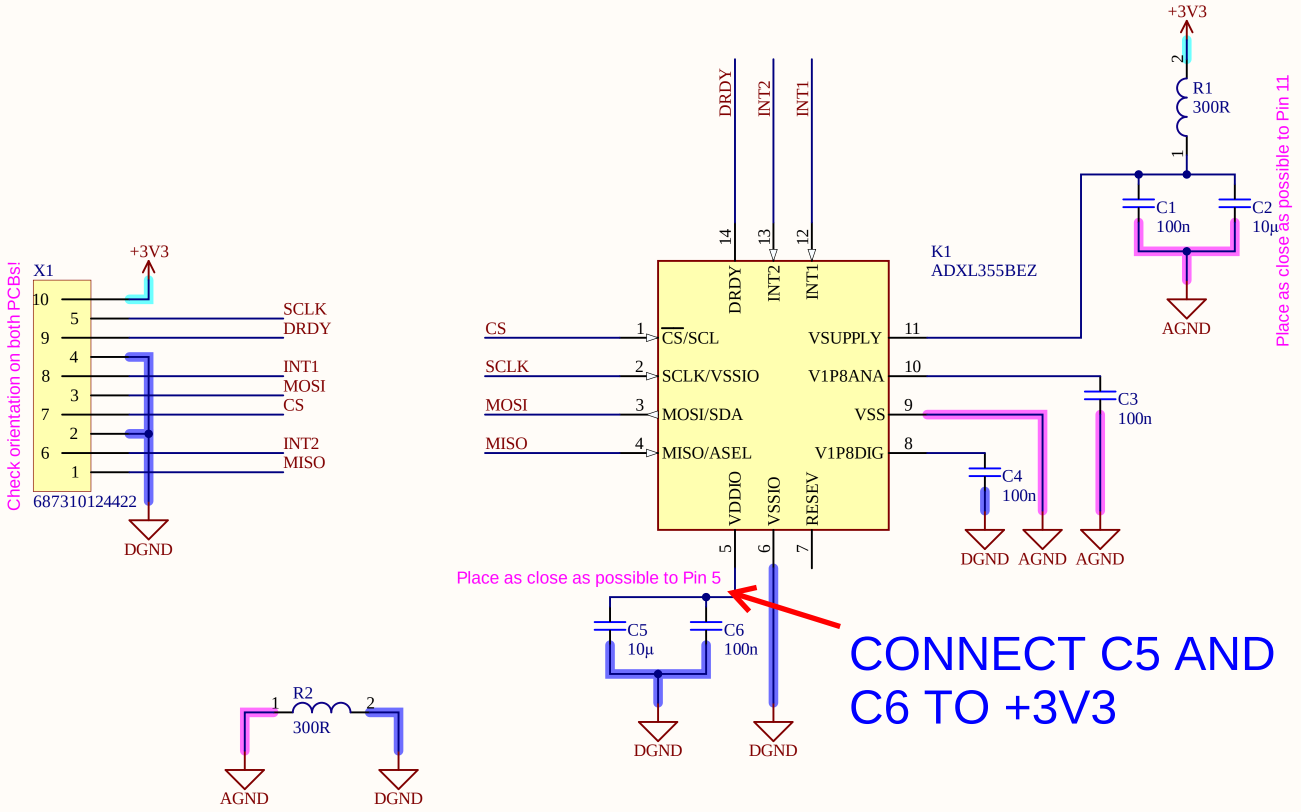

Project *AccelSens*

Module SENSOR_Board

Drawn by: Sandurkov

Approved by: *

Title **SENSOR_Board**

Munich Institute of Biomed. Eng.
Technische Universität München
AG Magnetische Felder
Boltzmannstrasse 11
85748 Garching

Size: A4 Number: 1 Revision: 1

Date: 12.04.23 Sheet 1 of 1

File: SENSOR_Board.SchDoc

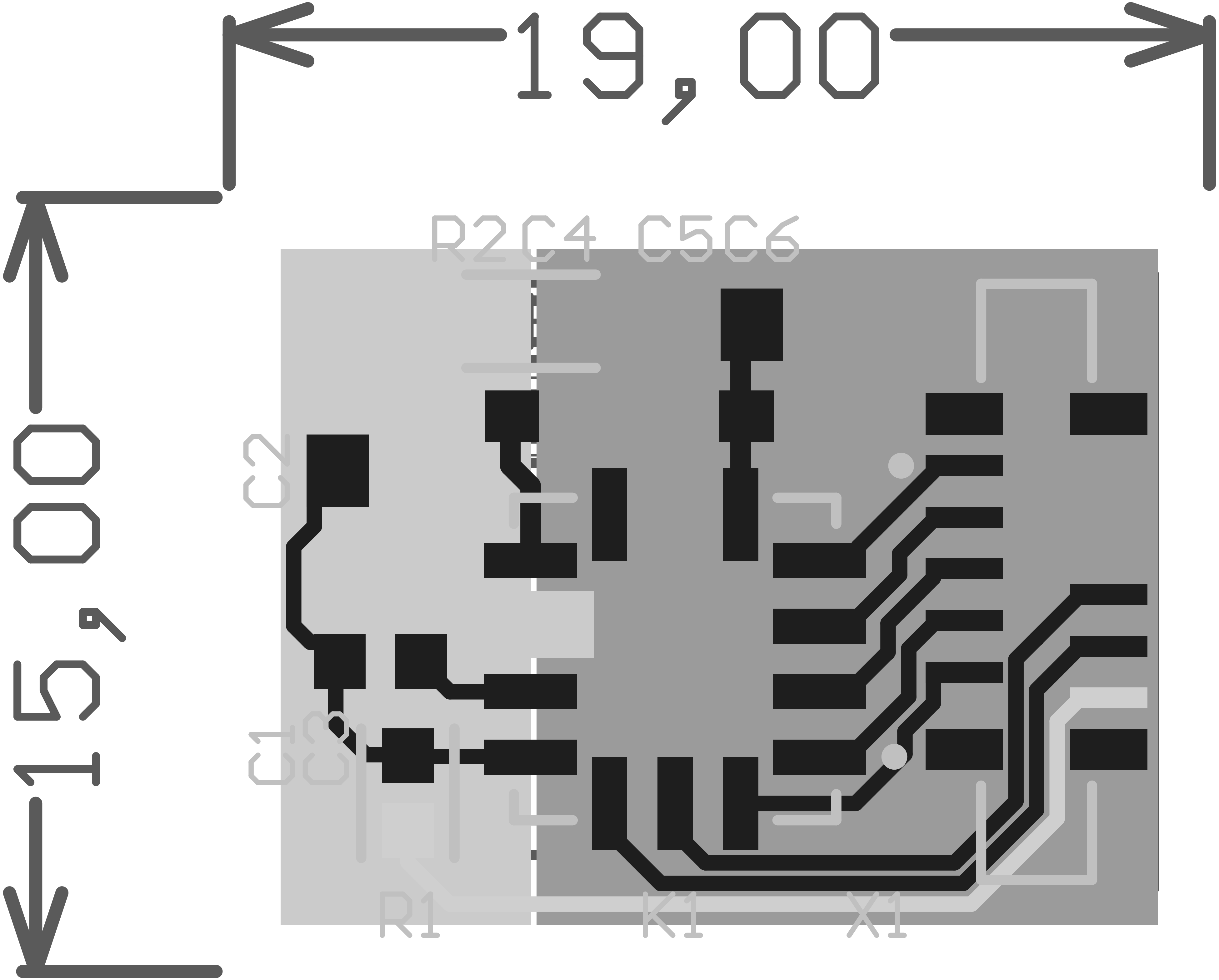

19,00
15,00
R2
C4
C5
C6
C2
C1
C3
R1
K1
X1
