# Supplementary material for: A compact setup for behavioral studies measuring limb acceleration
Source: HardwareX. 2024 Apr 6;18:e00522. doi: 10.1016/j.ohx.2024.e00522 (PMC11022083; doi:10.1016/j.ohx.2024.e00522)
Supplement: MMC S1 — The supplementary contains electrical schematics and layout for the sensor and controller board as well as the bills of material. [file mmc1.zip › OHX_522 - Supplementary files.zip!/OHX_522 - Supplementary files/Total_BOM.pdf]

Sensor Board

Designator	Component	Description	Quantity	Supplier	Currency	Unit Price	Total Price	
C1, C3, C4, C6	C0603C104K5RACTU	100n, 0603, X7R, 10%, 50 V	4	mouser.com	€	0,102	0,408	Electrical component
C2, C5	C0805C106K4PACTU	10µ, 0805, X5R, 10%, 16 V	2	mouser.com	€	0,149	0,298	Electrical component
K1	ADXL355BEZ	ACCELEROMETER2-4-8GDIGTL14QFN	1	mouser.com	€	68,38	68,38	Electrical component
R1, R2	742792641	300R / 100 MHz, 2 A, 0R15, WE-CBF	2	mouser.com	€	0,13	0,26	Electrical component
X1	687310124422	Ffc / Fpc Board Connector, Vertical, Surface Mount, Receptacle, 10, 0.5 Mm Rohs Compliant: Yes	1	mouser.com	€	1,63	1,63	Electrical component

Controller Board

Designator	Component	Description	Quantity	Supplier	Currency	Unit Price	Total Price	
B1	NX3225GD-8MHZ-STD-CRA-3	Crystal, 8 MHz, 8 pF, NX3225GD	1	mouser.com	€	0,809	0,809	Electrical component
C1, C6	C0603C829C5GACTU	8.2 pF, 50 V, 5%, C0G, 0805	2	farnell.com	€	0,119	0,238	Electrical component
C2, C8, C9, C13, C14, C15, C16, C19, C21, C23	C0603C104K5RACTU	100n, 0603, X7R, 10%, 50 V	10	farnell.com	€	0,0597	0,597	Electrical component
C3, C4	C0805C105K3RACTU	1 µF, 25 V, 10%, X7R, 0805	2	farnell.com	€	0,059	0,118	Electrical component
C5, C10	C0805C103K5RACTU	10 nF, 50 V, 10%, X7R, 0805	2	farnell.com	€	0,046	0,092	Electrical component
C7	CC0603KRX7R9BB103	10 nF, 50 V, 10%, X7R, 0603	1	farnell.com	€	0,053	0,053	Electrical component
C11, C12	C0805C475K4PACTU	4.7 µF, ± 10%, X5R, 16 V, 0805	2	digikey.com	€	0,121	0,242	Electrical component
C17, C22	C0805C106K4PACTU	10µ, 0805, X5R, 10%, 16 V	2	farnell.com	€	0,086	0,172	Electrical component
F1	STM32F401RET6	M4, 512 kB, 84 MHz	1	mouser.com	€	8,24	8,24	Electrical component
F2	UCLAMP0571P.TNT	TVS DIODE 5V 15V SGP1610N2	1	mouser.com	€	0,76	0,76	Electrical component
F3	WE-TVS-HS, 824015	TVS Diode WE-TVS-HS, VRWM=5V	1	mouser.com	€	0,837	0,837	Electrical component
K1	TPS73233DBVT	Low Noise, Positive Output LDO, 250 mA, 3.3V	1	mouser.com	€	1,81	1,81	Electrical component
K2	STM1061N29WX6F	Low Power Voltage Detector, Open Drain, 2,9 V Threshold, SOT23-3	1	mouser.com	€	0,939	0,939	Electrical component
K3	SN74AHC1G08DBVR	CMOS Single AND-Gate, AHC, 2 V - 5.5 V, 8 mA, Ultra High Speed	1	mouser.com	€	0,307	0,307	Electrical component
P1, P2	150060VS75000	WL-SMCW LED, green, 30 mA, 2 V, 570 nmr, 0603, Bright Green, 140°	2	mouser.com	€	0,14	0,28	Electrical component
R1, R3, R4, R5, R6, R7, R8, R16	CRCW0603100RFKEAHP	100R, 0603, 0.25 W, 1%, 75 V, pulse proof	8	mouser.com	€	0,13	1,04	Electrical component
R2, R10	CRCW06033K30FKEA	3k3, 0603, 0.1 W, 1%, 75 V	2	mouser.com	€	0,093	0,186	Electrical component
R9	CRCW06031K00FKEA	1k, 0603, 0.1 W, 1%, 75 V	1	mouser.com	€	0,093	0,093	Electrical component
R11	WE-CNSW 1206, 744232090	SMD Common Mode Noise Suppressor WE-CNSW, Z= 90 Ohm	1	mouser.com	€	1,19	1,19	Electrical component
R12, R17, R18, R19, R20, R21	CRCW060310K0FKEA, ERA3AEB103V	10k, 0603, 0.1 W, 1%, 75 V, 10k, 0603, 0.1 W, 0.1%, 75 V	6	mouser.com	€	0,093	0,558	Electrical component
R13, R14	CRCW06035K10FKEA	5k1, 0.1 W, 1%, 75 V, 0603	2	mouser.com	€	0,13	0,26	Electrical component
R15	742792641	300R / 100 MHz, 2 A, 0R15, WE-CBF	1	mouser.com	€	0,13	0,13	Electrical component

T1	PKLCS1212E4001-R1	SUMMER, SMD, 4KHZ EXTERN BESCHALTEN	1	mouser.com	€	0,96	0,96	Electrical component
X1	687310124422	Ffc / Fpc Board Connector, Vertical, Surface Mount, Receptacle, 10, 0.5 Mm Rohs Compliant: Yes	1	Mouser	€	1,63	1,63	Electrical component
X2	629722000214	USB 2.0 Type C	1	farnell.com	€	3,08	3,08	Electrical component
X3	WE-3.00 mm Micro Power Connector 66200221022	WR-MPC3 3.00 mm Male Dual Row Angled Header with Plastic Pegs	1	mouser.com	€	0,61	0,61	Electrical component
X4	TC2050-IDC	JTAG - Adapter Cbl Plug Of Nails 10PIN	1	digikey.com	€	36,99	36,99	Electrical component
**Glove**								
1	ESD fabric		30 × 30 cm^2	stoffe.de	€	8,99 €/m	8,99	fabric
2	Sport jersey		30 × 30 cm^2	tigerlilly.de	€	1,98 €/m	2,69	fabric
3	Bio Zwirn Doppelpilot		1	'tigerlilly.de	€	2,59	2,59	fabric
4	VELCRO® Veloursband loops 10 mm		30 cm	'tigerlilly.de	€	1,1	1,1	fabric
5	VELCRO® Velours band hook fastener 10 mm		30 cm	'tigerlilly.de	€	1,1	1,1	fabric
							149,667	
